# Neurotransmission-related gene expression in the frontal pole is altered in subjects with bipolar disorder and schizophrenia

**DOI:** 10.1038/s41398-023-02418-1

**Published:** 2023-04-08

**Authors:** Adriana M. Medina, Megan Hastings Hagenauer, David M. Krolewski, Evan Hughes, Liam Cannon Thew Forrester, David M. Walsh, Maria Waselus, Evelyn Richardson, Cortney A. Turner, P. Adolfo Sequeira, Preston M. Cartagena, Robert C. Thompson, Marquis P. Vawter, Blynn G. Bunney, Richard M. Myers, Jack D. Barchas, Francis S. Lee, Alan F. Schatzberg, William E. Bunney, Huda Akil, Stanley J. Watson

**Affiliations:** 1 Michigan Neuroscience Institute, University of Michigan, Ann Arbor, MI USA; 2 University of California-Irvine, Irvine, CA USA; 3 HudsonAlpha Institute for Biotechnology, Huntsville, AL USA; 4 Weill Cornell Medical College, New York, NY USA; 5 Stanford University, Palo Alto, CA USA

**Keywords:** Molecular neuroscience, Bipolar disorder, Genomics, Schizophrenia, Addiction

## Abstract

The frontal pole (Brodmann area 10, BA10) is the largest cytoarchitectonic region of the human cortex, performing complex integrative functions. BA10 undergoes intensive adolescent grey matter pruning prior to the age of onset for bipolar disorder (BP) and schizophrenia (SCHIZ), and its dysfunction is likely to underly aspects of their shared symptomology. In this study, we investigated the role of BA10 neurotransmission-related gene expression in BP and SCHIZ. We performed qPCR to measure the expression of 115 neurotransmission-related targets in control, BP, and SCHIZ postmortem samples (*n* = 72). We chose this method for its high sensitivity to detect low-level expression. We then strengthened our findings by performing a meta-analysis of publicly released BA10 microarray data (*n* = 101) and identified sources of convergence with our qPCR results. To improve interpretation, we leveraged the unusually large database of clinical metadata accompanying our samples to explore the relationship between BA10 gene expression, therapeutics, substances of abuse, and symptom profiles, and validated these findings with publicly available datasets. Using these convergent sources of evidence, we identified 20 neurotransmission-related genes that were differentially expressed in BP and SCHIZ in BA10. These results included a large diagnosis-related decrease in two important therapeutic targets with low levels of expression, HTR2B and DRD4, as well as other findings related to dopaminergic, GABAergic and astrocytic function. We also observed that therapeutics may produce a differential expression that opposes diagnosis effects. In contrast, substances of abuse showed similar effects on BA10 gene expression as BP and SCHIZ, potentially amplifying diagnosis-related dysregulation.

## Introduction

The frontal pole (Brodmann area 10, BA10) is the largest cytoarchitectonic region of the human cortex [1] and perhaps one of the most evolutionarily advanced. Relative to other non-human primates, the adult human BA10 occupies twice the proportion of the cortical extension and has much lower cellular density [2, 3]. BA10 has more dendritic spines per cell compared to adjoining areas [4] and extensive connections with higher-order association areas [5], suggesting high levels of input integration.

During adolescence, these connections are refined during an intensive pruning period, producing large decreases in BA10 cortical thickness [6]. This developmental pruning precedes the average age of onset of bipolar disorder (BP) and schizophrenia (SCHIZ) [7], and may be dysregulated in association with psychiatric illness [8]. By adulthood, individuals with SCHIZ and BP show reduced BA10 grey matter thickness compared to control (CTRL) subjects [8, 9], and reduced BA10 cortical surface area [9], especially in association with psychosis [8] and antipsychotic treatment [9]. Functionally, subjects with BP and SCHIZ have heightened BA10 resting-state activity [10, 11], as well as altered functional connectivity to sensory/association areas and subcortical regions [12, 13].

The role of BA10 dysfunction in psychiatric symptomology is unclear. BA10 is associated with complex cognitive functions that depend on maintaining mental representations of alternative courses of action [5], such as multitasking, prospective memory, time estimation, decision-making, task-switching, and flexible emotional control [1, 5]. Accordingly, BA10 abnormalities in SCHIZ and BP have been linked to deficits in working memory [11], cognitive control [14], and impulse control [15, 16]. BA10 also allows for the integration of internally- and externally obtained cognition [1], suggesting a potential role in psychotic symptoms, including delusions, hallucinations, and disorganized speech.

For this study, we examined neurotransmission-related gene expression in BA10 in subjects with BP and SCHIZ, due to its relevance to both disease etiology and the design of therapeutics [17–19]. We chose the highly sensitive method of qPCR, allowing us to confirm and expand on previous studies tackling the topic with gene expression profiling [20–24], and targeted 115 transcripts related to the main neurotransmitter systems in the frontal cortex: glutamate, GABA, dopamine, and serotonin [25]. We augmented our findings with a meta-analysis of publicly available microarray data [20, 21] and an in-depth examination of accompanying clinical characteristics to clarify the role of BA10 in the shared symptomology of the two disorders [26].

## Methods

This research was overseen and approved by the University of Michigan Institutional Review Board, Pritzker Neuropsychiatric Disorders Research Consortium, and the University of California-Irvine Institutional Review Board. Key Resources (Supplementary Table S1) and full methodological details are documented in the supplement (Supplemental Methods and Results).

Human samples were collected through the University of California-Irvine Pritzker Brain Donor Program with informed consent from next of kin (*n* = 72, CTRL: *n* = 27, BPD: *n* = 21, SCHIZ: *n* = 24; Supplementary Table S2 and Supplementary Fig. S1 [27]). The sample size was determined by the amount of tissue available surpassing strict quality metrics (agonal factor 0, pH >6.5), and should be adequate to detect large effect sizes (*d* > 0.8) with 80% power. A detailed psychological autopsy was performed using coroner records, medical records, and interviews with the next-of-kin (Supplemental Methods and Results 1). This information was used to confirm BP and SCHIZ diagnosis (criteria: [26]), and ensure the absence of neurological or psychiatric disorder in CTRL subjects or their first-degree relatives. Other clinical information was summarized as 49 exploratory variables (Supplementary Fig. S2) denoting the presence or absence of (1) medication, (2) exposure to alcohol or drugs of abuse, and (3) diagnosis-related symptoms.

Brains were extracted during autopsy and kept on ice until being sliced into 1 cm thick coronal slabs, then snap-frozen for storage (−80 °C) until microdissection. After counterbalancing processing batches by diagnosis, samples were blinded, and the foremost rostral slab from the left hemisphere was sub-dissected to obtain blocks averaging 500 µg containing lateral BA10 (Supplementary Fig. S3), a subregion implicated in SCHIZ [13]. RNA was extracted using TRIzol™ and purified (RNeasy® Mini Kit). cDNA was synthesized (iScript Reverse Transcription Supermix kit) and analyzed in duplicate via qPCR (Applied Biosystems ViiA 7 real-time PCR system) using two sets of ThermoFisher Scientific Taqman Gene Expression Array qPCR cards: (1) “Human GABA Glutamate” (REF#4342259): 84 targets (12 reference genes), (2) “Dopamine Serotonin” (REF#4342253): 31 targets (17 reference genes) (Supplementary Table S3). These cards were preloaded with a complete list of targets for the main neurotransmitter systems in the frontal cortex: glutamate, GABA, dopamine, and serotonin [25], including receptors, transporters, metabolic enzymes, and other associated molecules. The cards were further customized to include well-known markers for interneuron subtypes (SST, PVALB, CALB1) and astrocytes (AQP4, GJA1, GFAP, KCNJ10, S100B) to enhance the interpretation of neurotransmission-related data. qPCR quantification cycle (Cq) data for targets were normalized using the averaged reference gene expression for each sample to produce -ΔCq values [28]. Covariation within the replicate samples for each subject was accounted for during differential expression analysis using multilevel modeling [29, 30].

For comparison, we performed a meta-analysis of publicly released BA10 microarray data (re-annotated and re-analyzed: Gene Expression Omnibus #GSE12654 [20]: *n* = 50, CTRL: *n* = 15, BPD: *n* = 11, SCHIZ: *n* = 13, MDD = 11; #GSE17612 [21]: *n* = 51, CTRL: *n* = 23, SCHIZ: *n* = 28) using random effects modeling [31] of the Log(2) fold change (Log2FC) and sampling variance (standard error (SE) [2]) for each gene (EntrezID) from the *limma* [32] differential expression output for each dataset.

Both qPCR and microarray differential expression analyses controlled for common sources of biological and technical noise (*all:* pH, PMI, age, sex; *qPCR:* RIN, RNA concentration, Card; *microarray* (when applicable): RNA degradation, rate of death, scan date) and corrected for false discovery rate (FDR: Benjamini–Hochberg method [33]). All statistical tests were performed using two-sided *P* value calculations. When possible, findings were directly compared to relevant published datasets [23, 34–44] to determine validity and generalizability.

## Results

To evaluate the effects of BP and SCHIZ on neurotransmission-related gene expression in BA10 using highly sensitive methodology, we ran all samples in duplicate on two custom sets of Taqman qPCR cards loaded with 115 targets related to the main neurotransmitter systems in the frontal cortex [25]. We then performed a meta-analysis of previous BA10 microarray studies [20, 21] and used the convergence between our qPCR and microarray results to identify additional differentially expressed neurotransmission-related genes. To explore the function of these differentially expressed genes, we compared our findings across diagnosis categories, and explored the similarity between our findings and those from adjacent cortical areas. We then leveraged the extensive metadata accompanying our samples to explore the relationship between gene expression and clinical characteristics, using published data to bolster exploratory findings.

A summary of the main findings is presented below. Comprehensive details are in the Supplement, including all de-identifiable sample metadata (64 technical and clinical variables, Supplementary Table S2) and detailed statistical reporting to accompany the full qPCR and microarray results (Supplementary Figs. S4–S21 and Supplementary Tables). The full qPCR datasets (Cq), normalized results (-ΔCq), and deidentified metadata have been publicly released (10.6084/m9.figshare.20520378.v1bc).

### qPCR results

#### Balanced design

Tissue was collected from 72 subjects. Three subjects were later excluded due to poor RNA quality metrics, leaving *n* = 69 (CTRL: *n* = 26, BP: *n* = 21, SCHIZ: *n* = 22). Subjects were predominantly Caucasian (91%) and male (88%), although a quarter of the BP group was female (diagnosis vs. sex: *P* = 0.0174, Supplementary Fig. S5). The diagnosis groups were otherwise balanced for critical biological and technical variables (Supplementary Table S4 and Supplementary Fig. S5, *P* > 0.10: age, brain pH, postmortem interval (PMI), tissue block weight, RNA concentration, purity (260/280, 260/230), and integrity (RNA integrity number (RIN), 28 S/18 S). All subjects experienced a fast death (agonal factor score = 0). BP subjects with information regarding mood at the time of death were in a depressive state (*n* = 12).

#### Effect of diagnosis

We characterized the effect of diagnosis on the expression of 111 neurotransmission-related target genes (Supplementary Table S5) while controlling for sources of biological and technical noise (pH, PMI, age, sex, RIN, RNA concentration, card). Results were remarkably robust to model specification (Supplementary Fig. S12). As expected, qPCR accurately measured the expression of target genes with extremely low levels of expression (quantification cycles (Cq) between 27-34.6) without decreased accuracy (Supplementary Fig. S13A–C and Supplementary Table S3). This allowed us to accurately characterize the relationship between diagnosis and the expression of neurotransmission-related genes that were not reliably quantifiable within previous microarray and RNA-Seq studies (Supplementary Fig. S13D–G).

**Fig. 1:**
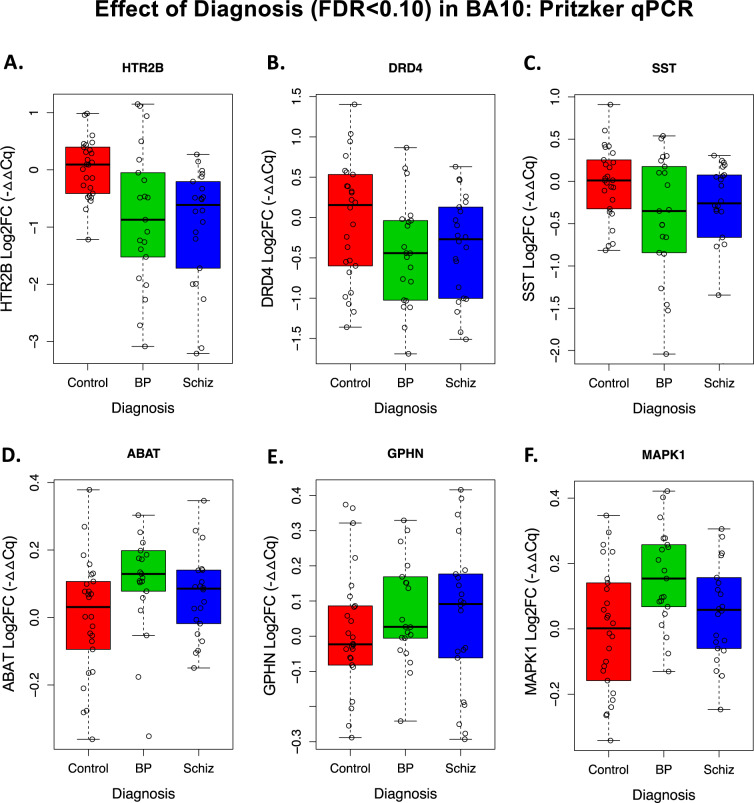
Six target genes showed a relationship with the diagnosis that survived false discovery rate correction (FDR < 0.10) in our BA10 qPCR experiment. Boxplots illustrate the distribution of −ΔΔCq values *(*log2 fold change (Log2FC), with the average for the CTRL group set as 0) for each subject within each diagnosis group (boxes=first quartile, median, and third quartile, whiskers = range and/or 1.5× the interquartile range if there are outlying data points). For ease of visualization, each datapoint represents an average of the individual replicates for each subject, without the additional correction for influential sources of biological variation (age, pH, sex) or technical variation (PMI, RIN, RNA Concentration, Card) provided by our multilevel statistical model (statistics reported below). **A** 5-Hydroxytryptamine Receptor 2B (HTR2B) showed an effect of diagnosis (*P* = 0.000312, FDR = 0.0173), with a decrease in both bipolar disorder (BP, Log2FC = -0.681, *P* = 0.0270) and schizophrenia (SCHIZ, Log2FC = -0.980, *P* = 0.000903). **B** Dopamine Receptor D4 (DRD4) showed an effect of diagnosis (*P* = 0.00196, FDR = 0.0495), with a decrease in both BP (Log2FC = -0.466, *P* = 0.00510) and SCHIZ (Log2FC = -0.347, *P* = 0.0244). **C** Somatostatin (SST) showed an effect of diagnosis (*P* = 0.00223, FDR = 0.0495), with a trend towards a decrease in BP (Log2FC = −0.308, *P* = 0.0935) and a decrease in SCHIZ (Log2FC = -0*.*467, *P* = 0.00689), **D** 4-aminobutyrate aminotransferase (ABAT) showed an effect of diagnosis (*P* = 0.00166, FDR = 0.0495), with elevation in BP (Log2FC = 0.157, *P* = 0.00550) and a trend towards elevation in SCHIZ (Log2FC = 0*.*083, *P* = 0.0993). **E** Gephyrin *(*GPHN) showed an effect of diagnosis (*P* = 0.000283, FDR = 0.0173), with elevation in BP (Log2FC = 0.156, *P* = 0.00256) and SCHIZ (Log2FC = 0*.*101, *P* = 0.0295). **F** Mitogen-activated protein kinase 1 (MAPK1) showed an effect of diagnosis (*P* = 0.00372, FDR = 0.0688), with elevation in BP (Log2FC = 0.135, *P* = 0.00113) and a trend towards elevation in SCHIZ (Log2FC = 0.0869, *P* = 0.0730). Complete statistical reporting for the full concatenated qPCR results can be found in Supplementary Table S5.

Indeed, two of our strongest findings were monoamine receptor genes with very low levels of cortical expression: 5-hydroxytryptamine Receptor 2B (HTR2B, average Cq: 31.8) and Dopamine Receptor D4 (DRD4, average Cq: 33.6). Both genes showed a relationship with the diagnosis that survived false discovery rate correction (FDR < 0.10) due to decreased expression in BP and SCHIZ (Fig. 1A, B). Notably, the magnitude of the decrease in HTR2B in SCHIZ was large—almost a full halving of normal expression levels.

Similar to previous studies, we found that the interneuron marker somatostatin (SST) showed a relationship with diagnosis (FDR < 0.10), with lower expression in BP and SCHIZ (Fig. 1C). Three other genes had elevated expression in both diagnosis groups: 4-aminobutyrate aminotransferase (GABA transaminase: ABAT), the postsynaptic scaffolding protein Gephyrin (GPHN), and the intracellular signaling molecule mitogen-activated protein kinase 1 (MAPK1) (Fig. 1D–F).

### Similarity to diagnosis effects in previous BA10 microarray studies

We compared our qPCR results to the published results from previous BA10 microarray studies of BP and SCHIZ [20–24] (Supplementary Table S7). To increase consistency, we re-annotated and re-analyzed the data from the two studies with publicly released data (Iwamoto et al. [20] and Maycox et al. [21]), and performed a meta-analysis of the SCHIZ effects (Log2FC) in the re-analyzed data.

**Fig. 2:**
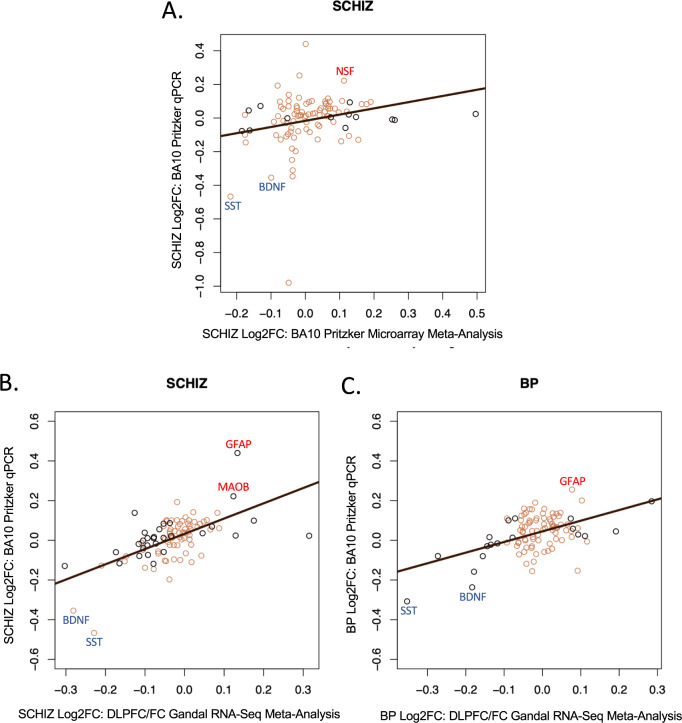
Our BA10 qPCR experiment replicated many diagnosis effects observed in the frontal cortex in meta-analyses of differential expression results from less sensitive transcriptional profiling methods (microarray, RNA-Seq). In addition to detecting novel diagnosis effects in low-level expressed genes (HTR2B, DRD4), our BA10 qPCR experiment was able to detect diagnosis effects that were observed in the frontal cortex within our current meta-analysis of previous BA10 microarray studies and in a previously-published meta-analysis of RNA-Seq studies of adjacent frontal cortex. **A** To provide a more robust comparison, we re-annotated and re-analyzed two publicly available BA10 microarray datasets (Iwamoto et al. [20] and Maycox et al. [21]) and then performed a meta-analysis to identify the differential expression associated with SCHIZ. A scatterplot is shown illustrating the positive correlation (*R* = 0.243, *P* = 0.0175) between the differential expression (Log2FC) associated with SCHIZ in our BA10 microarray meta-analysis and the differential expression associated with SCHIZ in our BA10 qPCR experiment (Log2FC) for all genes that were present in both datasets (*n* = 95). The color of the data points signifies whether a gene showed a nominally significant effect of diagnosis in the microarray meta-analysis results (black: *P* < 0.05, brown: *P* > 0.05). **B** A scatterplot illustrating the positive correlation (*R* = 0.607, *P* = 6.604e-11) between the differential expression associated with SCHIZ (Log2FC) identified in a large (*n* = 384: 203 CTRL, 181 SCHIZ) meta-analysis of RNA-Seq data from the DLPFC and other frontal cortical (FC) tissue (Gandal et al. [34]) and the differential expression associated with SCHIZ in our BA10 qPCR experiment (Log2FC) for all genes that were present in both datasets (95 genes). The color of the data points signifies whether a gene showed a nominally significant effect of diagnosis in the DLPFC/FC Gandal RNA-Seq meta-analysis results (black: *P* < 0.05, brown: *P* > 0.05). **C** A scatterplot illustrating the positive correlation (*R* = 0.490, *P* = 4.61e-07) between the differential expression associated with BP (Log2FC) identified in a large (*n* = 171: 101 CTRL, 70 BP) meta-analysis of RNA-Seq data from the DLPFC and other frontal cortical tissue (Gandal et al. [34]) and the differential expression associated with BP in our BA10 qPCR experiment (Log2FC) for all genes that were present in both datasets (95 genes). The color of the data points signifies whether a gene showed a nominally significant effect of diagnosis in the DLPFC/FC Gandal RNA-Seq meta-analysis results (black: *P* < 0.05, brown: *P* > 0.05). Data points are labeled with official gene symbols for genes that show particularly large Log2FC in both datasets. Full statistical reporting for the correlations between the differential expression observed in different datasets can be found in Supplementary Table S9.

**Fig. 3:**
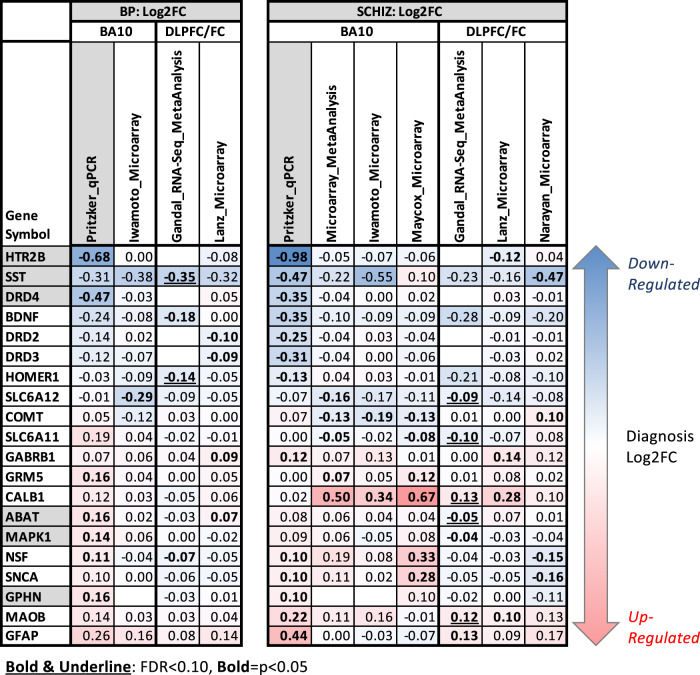
A table overviewing the most supported effects of diagnosis on neurotransmission-related gene expression in BA10. The table illustrates the effects of diagnosis (Log2FC, left: BP, right: SCHIZ) on neurotransmission-related gene expression as identified in our BA10 qPCR experiment (column highlighted in grey), our re-analysis of two BA10 microarray datasets (Iwamoto et al. [20] and Maycox et al. [20]), and our meta-analysis of the SCHIZ results from the two BA10 microarray datasets. These results often paralleled results from adjacent frontal cortex: a large meta-analysis of RNA-Seq data from the DLPFC/FC (Gandal et al. [34]) and results from two smaller microarray datasets from the DLPFC with grey matter-focused dissections (Lanz et al. [36] and Narayan et al. [35] as re-analyzed by Hagenauer et al. [37]). Note that we did not include the results from the large Gandal et al. [34] meta-analysis of microarray data because it was from a more broadly defined anatomical region (“cortex”) and included data from the Maycox et al. [21], Lanz et al. [36], and Narayan et al. [35] studies already in the table. To be included in the table, a gene needed to show strong evidence of differential expression in BA10: either FDR < 0.10 in our BA10 qPCR experiment (gene symbol with grey shading) or *P* < 0.05 (Log2FC in bold text) and consistent direction of effect in two independent datasets from BA10 or in a BA10 dataset and DLPFC/FC dataset. Throughout the table, red is used to indicate upregulation and blue is used to indicate downregulation. Empty cells indicate that the expression for that gene was either not quantified (GPHN) or considered to be unquantifiable (HTR2B, DRD2/3/4) in the dataset. Complete statistical reporting for the full results from our BA10 qPCR experiments, our re-analysis of the Iwamoto et al. and Maycox et al. BA10 microarray datasets and BA10 microarray meta-analysis can be found in Supplementary Tables S5 and S8.

In general, despite the noise in the microarray data and lack of sensitivity for low-level expression (Supplementary Fig. S13F, G, e.g., HTR2B, DRD4), we observed a similar direction of effect in the BA10 microarray datasets for top differentially expressed genes from our qPCR study (SST, ABAT, MAPK1, GPHN). We also observed a weak positive correlation between our full qPCR results and the re-analyzed BA10 microarray results (Fig. 2A and Supplementary Fig. S14, *qPCR* vs. *Iwamoto: SCHIZ:* *R* = 0.24, *P* = 0.0179; *BP:* *R* = 0.15, *P* = 0.151: *qPCR* vs. *Maycox: SCHIZ:* *R* = 0.09, *P* = 0.338*not sig; *qPCR* vs. *Meta-analysis: SCHIZ:* *R* = 0.24, *P* = 0.0175). Among these results, we identified three additional genes with nominally significant differential expression (*P* < 0.05) in both the qPCR study and BA10 microarray data as well as a consistent direction of effect (GRM5, NSF, SNCA, Fig. 3). Our meta-analysis also identified six genes differentially expressed with SCHIZ (FDR < 0.10) that were not included as qPCR targets (downregulated: TUBB7P, TIE1, HSD17B8, URM1; upregulated: CACYBP, GIT2, Supplementary Fig. S16, and Supplementary Table S8).

### Similarity to diagnosis effects in adjacent cortical regions

**Fig. 4:**
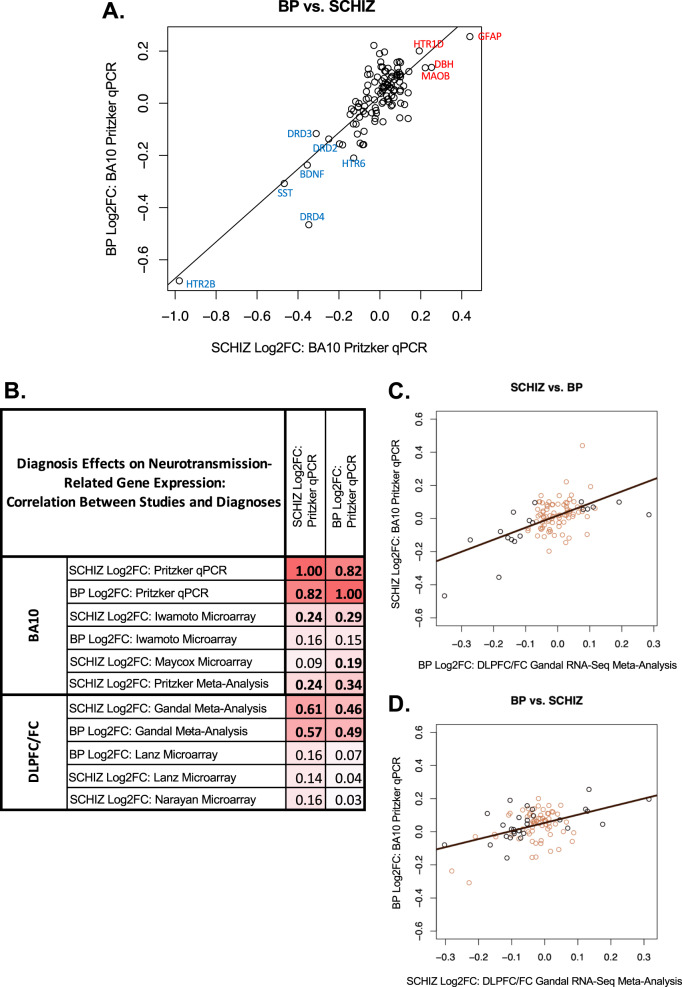
Cross-diagnosis comparison: BP and SCHIZ have similar differential expression in BA10 and adjacent cortex. **A** A scatterplot illustrating the correlation between the differential expression (Log2FC) for BP vs. SCHIZ for the 111 target genes included in our two BA10 qPCR datasets. In general, there is a strong positive correlation between the effects of the two diagnoses (*R* = 0.82, *P* < 2e-16). Data points are labeled with official gene symbols for individual genes with particularly large Log2FC for both diagnoses. **B** A table of correlation coefficients illustrating the similarity between the differential expression (Log2FC) associated with BP and SCHIZ in BA10 in our qPCR study and the differential expression (Log2FC) associated with both BP and SCHIZ in BA10 as measured by microarray (re-analyzed by our laboratory: Iwamoto et al. [20] and Maycox et al. datasets [21]) and in the DLPFC/FC as indicated by the large Gandal et al. meta-analysis of RNA-Seq studies [34] or by two smaller DLPFC microarray studies that used grey matter-focused dissections (Lanz et al. [36] and Narayan et al. [35]). Bold text indicates significance (*P* < 0.05). **C**, **D** Scatterplots illustrating the cross-diagnosis correlation between the effects of BP and SCHIZ within our BA10 qPCR dataset (Log2FC) and the effects of BP and SCHIZ within the results from the large DLPFC/FC Gandal et al. RNA-Seq meta-analysis [34] (Log2FC) for all genes that were present in both datasets (95 genes). The color of the data points signifies whether a gene showed a nominally significant effect of diagnosis in the DLPFC/FC Gandal et al. RNA-Seq meta-analysis results (black: *P* < 0.05, brown: *P* > 0.05). These findings indicate that the similarity between the effects of BP and SCHIZ within our BA10 qPCR dataset is not just an artifact due to using the same CTRL group as a reference in our analysis, but a valid property of the diagnosis-related differential expression. Full statistical reporting for the correlations between the differential expression observed in association with different diagnoses and different datasets can be found in Supplementary Table S9.

We observed a strong positive correlation between the diagnosis effects identified in our qPCR study and those previously observed in the dorsolateral prefrontal cortex (DLPFC) and adjacent frontal cortex (FC) in a large meta-analysis of RNA-Seq studies (Gandal et al. [34], Fig. 2B, C, *n* = Log2FC for 95 genes: SCHIZ: *R* = 0.61, *P* = 6.604e-11, BP: *R* = 0.49, *P* = 4.61e-07). As RNA-Seq still lacked sensitivity for measuring low-level expression (Supplementary Fig. S13D, E), the strength of this correlation likely reflected the statistical power of the RNA-Seq meta-analysis (*SCHIZ* vs. *CTRL: n* = 384*; BP* vs. *CTRL:* *n* = 171). A comparison of our qPCR findings to smaller DLPFC microarray studies (Lanz et al. [36] and Narayan et al. [35], re-analyzed in ref. [37]), produced weaker positive correlations (all *P* > 0.10: Fig. 4B and Supplementary Table S9).

Altogether, there were 13 neurotransmission-related genes that showed at least nominally significant (*P* < 0.05) diagnosis-related effects in BA10 within either our qPCR experiment or the re-analyzed BA10 microarray data, as well as similar effects (*P* < 0.05, consistent direction) within adjacent cortex (DLPFC/FC; Fig. 3). Adding these findings to our existing list of genes with strong evidence of differential expression in BA10 (FDR < 0.10 in our qPCR experiment or *P* < 0.05 and consistent direction of effect in two independent BA10 datasets) produced twenty strong candidates for follow-up analysis (Fig. 3).

### The similarity of neurotransmission-related differential expression across diagnosis categories

BP and SCHIZ showed similar effects on gene expression when examining results across all 111 target genes in our qPCR analysis (Fig. 4A*,* BP vs. SCHIZ: *R* = 0.82, *P* < 2e-16). We also observed a strong correlation between the effects of BP and SCHIZ on neurotransmission-related gene expression within the re-analyzed BA10 microarray data (*Iwamoto:* *R* = 0.43, *P* = 1.32e-05) and within results from adjacent frontal cortex (*Gandal RNA-Seq meta-analysis* [34]: *R* = 0.80, *P* < 2e-16; *Lanz Microarray* [36]: *R* = 0.82, *P* < 2.2e-16, Supplementary Table S9).

Alone, the strong correlations between the effects of different diagnoses within individual datasets are difficult to interpret, because diagnosis groups are typically compared to a shared control group. However, when comparing across datasets, these correlations become more compelling (Fig 4B–D). For example, the correlation between the SCHIZ effects within our qPCR dataset and the BP effects within the Gandal RNA-Seq meta-analysis [34] was almost as large as comparing the SCHIZ effects within our qPCR dataset to SCHIZ effects within the RNA-Seq meta-analysis (*SCHIZ* vs*. BP:* *R* = 0.57, *P* = 2.39e-09). A similarly strong correlation was observed when comparing BP effects in our qPCR study to SCHIZ effects in the RNA-Seq meta-analysis (*BP* vs. *SCHIZ:* *R* = 0.46, *P* = 3.39e-06).

These results suggest that the differential expression associated with BP and SCHIZ in BA10 may reflect symptoms, risk factors, or experiences common to both disorders.

### Exploratory analyses: factors contributing to diagnosis-related gene expression

To further explore the function of our top diagnosis-related genes (*n* = 20, Fig. 3), we examined the relationship between gene expression in our qPCR dataset and a rich database of 49 clinical characteristics compiled via in-depth psychological autopsy (Supplementary Fig. S2). We evaluated the effect of these clinical variables on gene expression while controlling for diagnosis and relevant biological and technical covariates. Exploratory results with independent validation or support are highlighted below (details in Supplementary Methods and Results).

### Overlap between the effects of diagnosis and common therapeutics

One question that typically arises while interpreting diagnosis effects within human postmortem studies is whether the observed effects are due to the illness or its treatment. Indeed, there were a large number of documented effects (FDR < 0.05) of therapeutics on the expression of our top 20 diagnosis-related genes within the comprehensive Drug Gene Budger database (https://maayanlab.cloud/DGB/ [39]), including 169 antipsychotic effects and 28 antidepressant effects from a variety of experiments. Given this precedent, we explored the effect of exposure to therapeutics in our qPCR dataset while controlling for diagnosis. Although our analyses were underpowered (*antipsychotics:* *n* = 9; *antidepressants:* *n* = 8), we observed some suggestive results that were bolstered by previous evidence (Supplementary Fig. S17A). MAOB was decreased with antidepressants (FDR < 0.10), paralleling documented functional inhibition [45, 46]. We also saw nominally (*P* < 0.05) decreased GPHN with antidepressants, and increased COMT and BDNF with antipsychotics that mirrored the effects in Drug Gene Budger [39]. Notably, antipsychotic effects tended to be in the opposite direction of diagnosis (Supplementary Fig. S17B–D). In contrast, the diagnosis-related increase in SNCA could be an artifact of therapeutics, as we found a nominal (*P* < 0.05) increase with antidepressants, and substantial documented upregulation with both antidepressants and antipsychotics (19 effects: Drug Gene Budger [38]).

### Overlap between the effects of diagnosis and substances of abuse

Similar to many psychiatric studies, our diagnosis groups contained an elevated rate of documented dependence on alcohol and tobacco as well as the use of other substances of abuse around the time of death (cannabinoids, opioids, stimulants, Supplementary Fig. S18A, B), whether due to therapeutic use, recreation, or substance use disorder. This elevated rate of substance use could partially reflect better documentation for psychiatric subjects, as toxicology analysis was performed more often (CTRL: 12%, SCHIZ: 43%, BP: 36%) due to the manner of death, but the high rate of overdose (SCHIZ: 23%, BP: 52%) suggested otherwise.

**Fig. 5:**
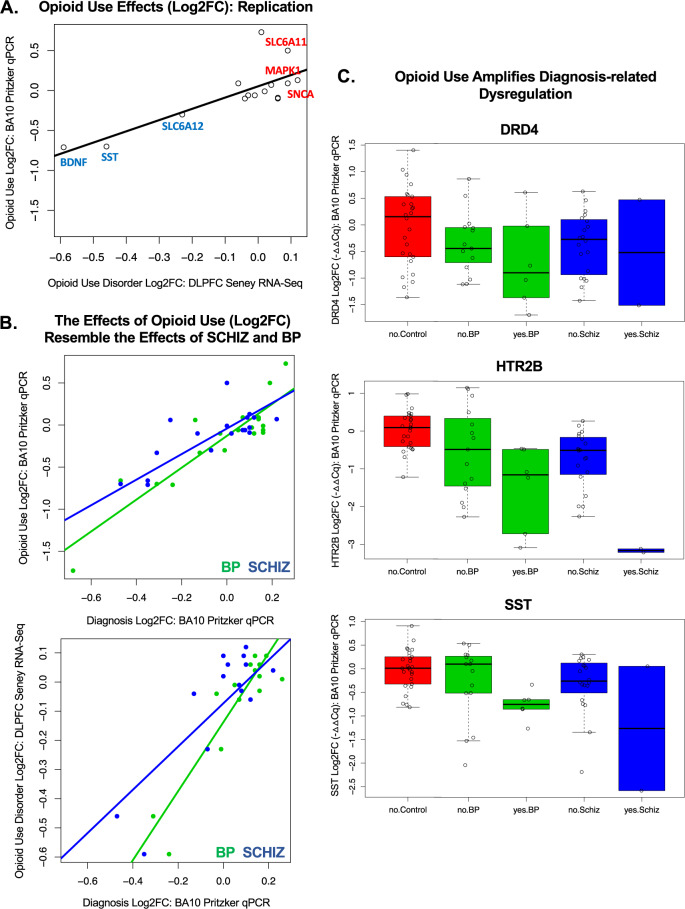
Exploratory: substances of abuse may be associated with similar dysregulation of neurotransmission-related gene expression in BA10 as SCHIZ and BP. Within a set of exploratory analyses, we estimated the differential expression (Log2FC) in BA10 in our qPCR dataset associated with a variety of substances of abuse (tobacco, cannabinoids, stimulants, opioids) while controlling for diagnosis. Substance exposure was defined by indication of usage within the subjects’ clinical records, family interviews, toxicology reports, and coroners reports. To reduce false discovery due to multiple comparisons, this exploratory analysis was limited to the 20 genes with the most reliable diagnosis effects in BA10 (listed in Fig. 3). The pattern revealed by our exploratory analysis was consistent across categories of substances of abuse (Supplementary Fig. S18D), but the largest effects were observed with opioid exposure (*n* = 8 subjects). **A** Replication: opioid exposure in our BA10 qPCR dataset was associated with similar differential expression (Log2FC) to what had been observed previously in opioid use disorder in the DLPFC using RNA-Seq by Seney et al. [41] (*n* = 15 diagnosis-related genes, BA10 Pritzker qPCR vs. DLPFC Seney RNA-Seq: *R* = 0.79, *P* = 0.000499). **B** Relationship with diagnosis: In general, the effects of diagnosis on gene expression (Log2FC) in BA10 measured in our qPCR study correlated positively with the effects of a variety of substances of abuse (Log2FC; tobacco, cannabinoids, stimulants, opioids) as estimated while controlling for diagnosis in our dataset. The top scatterplot uses opioid exposure as an example to illustrate the similarity between the effects associated with substances of abuse and diagnosis in our BA10 qPCR dataset (*n* = 20 diagnosis-related genes, *x* axis: Blue = BP Log2FC, Green = SCHIZ Log2FC, opioid Log2FC vs.: BP: *R* = 0.90, *P* = 5.04e-08, SCHIZ: *R* = 0.91, *P* = 2.21e-08). The bottom scatterplot shows that the similarity between the effects of opioid use and diagnosis replicates when using the effects of opioid use disorder measured in the DLPFC Seney et al. [41]. RNA-Seq dataset (*n* = 15 diagnosis-related genes, opioid use disorder Log2FC vs.: BP: *R* = 0.89, *P* = 6.43e-06, SCHIZ: *R* = 0.76, *P* = 0.000678). **C** Opioid use appears to amplify diagnosis-related dysregulation in our BA10 qPCR dataset: Example boxplots illustrate the effect of opioid exposure within our diagnosis groups for top diagnosis-related genes (DRD4: effect of diagnosis (*P* = 0.0190), effect of opioids (*P* = 0.000208); HTR2B: effect of diagnosis (*P* = 9.94e-05), effect of opioids (*P* = 9.42e-09); SST: effect of diagnosis (*P* = 0.0111), effect of opioids (*P* = 0.000146)). Plotting conventions follow Fig. 1, with “yes” and “no” indicating exposure to opioids. Differential expression statistics are derived from the multilevel model used previously, but with opioid use added as a predictor. Differential expression results for other genes and substances of abuse can be found in Supplementary Fig. S19. Full statistical reporting for correlations can be found in Supplementary Table S9.

Therefore, we explored whether diagnosis effects in our qPCR dataset could be better explained by substance use. For our top 20 diagnosis-related genes, we examined the effect of recent exposure to substances of abuse while controlling for diagnosis. We found that the effects (Log2FC) were consistent across substance categories (median: *R* = 0.77, range: *R* = −0.06–0.88, Supplementary Fig. S18D), in a manner that exceeded the correlated use of those substances (median: *R* = 0.44, range: *R* = 0.068–0.52, Supplementary Fig. S18C), suggesting that the results might broadly reflect use behaviors. This was further supported by the similarity between our results and previously documented effects of opioid use disorder (Seney et al. RNA-Seq [41]: *R* = 0.26–0.79, median *R* = 0.58, Fig. 5A and Supplementary Fig. S18D) and alcohol abuse disorder in the cortex (microarray meta-analysis [34] and RNA-Seq [40]: *R* = −0.03–0.69, median *R* = 0.36, Supplementary Fig. S18D), but we did not see a similar parallel with the effects of substance use in the smaller BA10 Iwamoto microarray dataset (Supplementary Fig. S18D, smoking, heavy alcohol, or heavy drug use).

For several genes, the effects of substances of abuse were large (FDR < 0.10) and consistent with evidence from larger studies in the adjacent frontal cortex (alcohol abuse disorder [34, 40], opioid use disorder [41]; Supplementary Fig. S19). GFAP was increased with a variety of substances (tobacco, simulants, opioids, overdose). HTR2B, SST, and BDNF were decreased with opioid use (Fig. 5C), overdose (HTR2B, SST), and nominally other substances of abuse. MAPK1 and GABRB1 were increased with stimulant use and nominally other substances of abuse. We also observed decreased DRD4 and SLC6A12 and increased SLC6A11 with substance use (FDR < 0.10, Fig. 5C and Supplementary Fig. S19) but lacked independent validation.

Notably, the effects of substances of abuse broadly resembled the effects of diagnosis (Fig. 5B, Supplementary Table S9, all relationships *R* = 0.54–0.90, *P* < 0.015), but diagnosis effects typically persisted after controlling for substance use (Fig. 5C and Supplementary Fig. S20). These exploratory findings are provocative, suggesting a mechanism by which substance use might converge with diagnosis-related dysregulation in frontal cortical function, but require further validation.

### Overlap between the effects of diagnosis, symptom profiles, and related behaviors

In general, there were very few symptoms and related behaviors in our dataset that had significant effects (FDR < 0.10) that resembled what we had observed for diagnosis. The relationships that did resemble the effects of diagnosis were often broadly related to executive dysfunction, with several genes differentially expressed in association with the terms “agitated” (SNCA, MAPK1, SLC6A12, HTR2B), “trouble concentrating” (GPHN), and indications of impulsivity (“reckless” (NSF), “interactions with the legal system” (GABRB1)). BDNF was decreased with “fatigue”, which can aggravate executive dysfunction (Supplementary Fig. S21A, B and Supplementary Table S9). These results are provocative, especially since some of these genes were also differentially expressed in the cortex in other diagnoses (Supplementary Fig. S21A, C) or in response to stress hormones (Supplementary Fig. S21A, D), but since the exploratory analyses were run while controlling for diagnosis, we may have had limited power to detect relationships with symptoms that more canonically define BP and SCHIZ, such as psychosis and disturbed affect. Further validation is strongly recommended.

## Discussion

Using the highly sensitive method of qPCR, we identified a large downregulation in BP and SCHIZ of two important therapeutic targets with low levels of expression in BA10, HTR2B, and DRD4, and detected weaker downregulation of DRD2 and DRD3. Our qPCR study also highlighted the differential expression of genes related to GABAergic and glutamatergic signaling, including SST, ABAT, GPHN, and MAPK1. To bolster our findings, we performed a meta-analysis of publicly released BA10 microarray data [20, 21], and identified sources of convergence with our qPCR results. We then compared our BA10 results to previous transcriptional profiling experiments in the adjacent cortex [34–36] and found many similar effects. Using this converging evidence, we identified twenty neurotransmission-related genes that were differentially expressed in BP and SCHIZ in BA10.

This differential expression was often similar across diagnoses, suggesting that gene expression associated with BP and SCHIZ in BA10 may reflect symptoms, risk factors, or experiences common across the two disorders. To explore factors contributing to diagnosis-related differential expression, we leveraged the extensive clinical metadata accompanying our qPCR samples to explore the relationship between BA10 gene expression, therapeutics, substances of abuse, and symptom profiles, and then validated exploratory findings with publicly available datasets. We found evidence that therapeutics may produce a differential expression that often opposes diagnosis effects. We also observed that substances of abuse may be associated with similar effects on gene expression in the frontal cortex as BP and SCHIZ, potentially amplifying diagnosis-related dysregulation.

### Downregulation of serotonin receptor HTR2B in BP and SCHIZ

The largest diagnosis effect in our study was a strong downregulation of the serotonin receptor HTR2B. Subjects with BP and SCHIZ had almost half as much HTR2B expression in BA10 as control subjects. We infer that this expression is probably located in vasculature and microglia based on previous studies [47–49]. If so, downregulation may impact two key features of SCHIZ and BP observed in the frontal lobe.

First, SCHIZ and BP symptoms correlate with altered patterns of blood flow in BA10 [11, 14, 16]. HTR2B is expressed in endothelial cells of the cerebral vasculature [49, 50] and regulates blood flow via the release of nitric oxide [47, 51]. Therefore, we hypothesize that HTR2B downregulation could contribute to altered BA10 blood flow.

Second, during adolescence, the frontal cortex experiences intense pruning that reduces grey matter thickness [6]. This pruning period precedes the onset of BP and SCHIZ [7] and may be disrupted in psychiatric illness [8]. Since microglia actively engulf synaptic structures during postnatal development, microglial dysfunction could contribute to aberrant pruning [52]. Microglia express HTR2B [48], and a lack of HTR2B receptors affects microglial activation and impedes their ability to mediate proper synaptic refinement [53]. These findings suggest that an HTR2B deficit could produce defective grey matter pruning in psychiatric subjects. Conversely, a diagnosis-related decrease in microglia could also produce an overall decrease in HTR2B expression.

In support of the hypothesis that decreased HTR2B contributes to the development of the disorders, previous exome sequencing studies have identified genetic variants associated with SCHIZ and BP within the HTR2B gene that may disrupt expression (missense/damaging, stop-gained [54–56]). HTR2B knock-out mice also exhibit an antipsychotic-sensitive behavioral phenotype reminiscent of SCHIZ, including decreased pre-pulse inhibition, dysfunctional social interaction, and cognitive deficits [57]. Acute exposure to the HTR2B antagonist RS127445 produces similar deficits [57].

Within our exploratory results, we also observed decreased HTR2B expression associated with substances of abuse, especially opioids, and the symptom of agitation. These findings are intriguing, since stop-gained genetic variants within the HTR2B gene are associated with impulsive behavior, including impulsive aggression, especially under the influence of alcohol [55, 58]. HTR2B knock-out mice are also more impulsive and responsive to novelty [55] and amphetamine [57], whereas, paradoxically, HTR2B antagonists can decrease behavioral responses to many drugs of abuse, including hyperactivity and conditioned place preference [59]. Collectively, these findings suggest that HTR2B deficits could affect impulsivity and drug-taking behavior, offering one explanation for the common covariation of substance abuse with BP and SCHIZ.

### Dopaminergic gene expression in BP and SCHIZ

Dopamine availability is pivotal to frontal cortical function [60–62] and theorized to play a role in both SCHIZ and BP. Disturbed dopamine function is important for psychosis, and most antipsychotic treatments target DRD2-like dopamine receptors (DRD2, DRD3, DRD4) [19]. In BP, failed homeostatic regulation of dopamine is theorized to underly mood cyclicity [63]. Genetic variation near dopamine receptor genes has been implicated in SCHIZ and BP by genome-wide association (DRD2);[64] linkage and association studies (DRD2, DRD3, DRD4) [65–68], and rare missense or stop-gained variants (DRD2 [69, 70], DRD3 [54], DRD4 [71]), implying that disrupted dopamine function may contribute to the disorders.

However, the relationship between SCHIZ and BP and the expression of DRD2-like receptors has been previously difficult to characterize due to low levels of expression in the frontal cortex [19, 72]. Early attempts using PCR produced mixed results, including in BA10 [73–75], although in situ hybridization detected region-specific, large-magnitude decreases in DRD4 and DRD3 in SCHIZ in nearby BA11 [76]. Using more modern qPCR methods, DRD2 was later found to be decreased in SCHIZ and increased in BP in the adjacent DLPFC [19]. We show that current qPCR methodology can reliably measure very low levels of expression, including the expression of DRD2-like dopamine receptors, and reveals large diagnosis effects in BA10 that dwarf those previously measured with less sensitive methodology (microarray, RNA-Seq).

Of the dopamine receptors, DRD4 showed the largest decrease in BP and SCHIZ in our dataset. Decreased DRD4 receptor activity could contribute to diagnosis-related disruptions in excitatory/inhibitory balance. An altered excitatory/inhibitory balance in areas associated with cortical inhibitory control is hypothesized to be a key feature of psychiatric illnesses with severe social and cognitive deficits [77, 78]. DRD4 agonists and antagonists decrease and increase postsynaptic GABA(A) receptor currents, respectively [79], and decreased DRD4 responsiveness is associated with decreased cortical hemodynamic activity [80]. In mice, DRD4 knockout causes disrupted GABAergic activity, which facilitates the development of SCHIZ-like symptoms under stress [81]. Moreover, the atypical antipsychotic clozapine has a high affinity for DRD4, and DRD4 knock-out mice are less responsive to its effects [82], suggesting that decreased DRD4 in BP and SCHIZ might not only contribute to disease etiology but also hinder successful treatment.

During our exploratory analyses, we observed decreased DRD4 in association with substances of abuse but were unable to find validation in an independent dataset. That said, DRD4 knock-out mice show an enhanced response to ethanol, cocaine, and methamphetamine [82], and DRD4 genetic polymorphisms have been implicated in substance use and executive dysfunction [83]. Therefore, similar to HTR2B, decreased DRD4 might promote the common covariation of substance abuse with BP and SCHIZ.

The enzyme catechol-O-methyltransferase (COMT) has been theorized to play a role in psychiatric disorders [61, 84–86]. Termination of monoamine activity in the frontal cortex depends mostly on the activity of COMT instead of the dopamine transporter [87]. Genetic variation near COMT has been potentially implicated in SCHIZ and BP by copy number variation [88], meta-analyses of linkage [67, 68] and association studies [65, 66], and rare variants [70]. We observed a nominal decrease in COMT in both BA10 microarray datasets that we re-analyzed. This downregulation might increase dopamine levels [61], with consequences such as aggression and increased stress sensitivity [87]. There was not a similar diagnosis effect in our qPCR dataset, but COMT was increased with antipsychotic treatment in a manner paralleling previous observations from cell culture and other tissues [39].

### GABA-related gene expression in BP and SCHIZ

Similar to our current work, previous studies in adjacent frontal areas have repeatedly shown large decreases in somatostatin (SST) in BP and SCHIZ ([34, 37, 89–91], review: [92]). SST is selectively expressed in GABAergic interneurons which represent ~30% of the total cortical interneuron population [93]. SST neurons synapse on pyramidal cell dendrites, indicating a role in filtering excitatory glutamatergic inputs [94]. Reduced SST interneuron function in SCHIZ is theorized to disrupt cortical information integration processes, such as working memory [92]. That said, decreased SST is not specific to psychotic disorders, and has been observed in Major Depressive Disorder (Supplementary Fig. S21) and following chronic stress [95]. SST neurons in the frontal cortex modulate anxiety-like behavior, and SST knock-out mice display elevated anxiety and corticosterone levels [95]. Both postmortem and preclinical studies link SST to drug-taking behavior [41, 95], which is supported by the decreased SST observed with substances of abuse in our exploratory analyses. Altogether, SST appears to be a compelling convergence point for stress to aggravate a variety of psychiatric symptoms.

Both BP and SCHIZ exhibited increased Gephryin (GPHN), a postsynaptic scaffolding protein which is abundant in the human cortex and localized to cell bodies and the apical dendrites of pyramidal neurons [96]. Rare genetic variants in GPHN have been associated with BP [70, 97]. GPHN helps cluster, anchor, and stabilize GABA(A) and glycine receptors at inhibitory synapses [98]. Therefore, the upregulation of GPHN in BP and SCHIZ could be related to the shift in GABA(A) receptor subunit composition that occurs at postsynaptic sites [99, 100], potentially contributing to increased GABA(A) receptor binding in the frontal cortex [100, 101]. This change in GABA(A) receptor binding, as well as the observed increased GABA(A) Receptor Subunit Beta1 (GABRB1) expression, might serve as a compensatory mechanism for disturbed local GABAergic neurotransmission.

### Astrocyte-related gene expression in BP and SCHIZ

We observed a large upregulation of the intermediate filament Glial fibrillary acidic protein (GFAP) in SCHIZ, an effect previously observed in the adjacent frontal cortex [34]. Increased expression of GFAP is a hallmark of astrocyte activation [102, 103] and follows increased intracellular calcium signaling [104]. Astrocyte reactivity is found in many pathological states involving excitotoxicity [102, 103], but we found little evidence of diagnosis-related glutamatergic alterations in BA10, except a nominal elevation of Glutamate metabotropic receptor 5 (GRM5). Instead, we hypothesize that astrocyte activation elicited by GABAergic stimulation might be a feature of BP and SCHIZ.

GABA is generally considered an inhibitory neurotransmitter, but it can elicit intracellular calcium increases and regional excitation in the glial syncytium [105, 106]. This increase in astrocytic calcium can be elicited by GABA(A) or GABA(B) receptor activation [107, 108]. Therefore, our results showing increased GABRB1 receptor subunit expression in SCHIZ could suggest a mechanism for increased astrocyte activation. We also found increased Monoamine oxidase B (MAOB) in both disorders. This enzyme synthesizes GABA in astrocytes [109]. GFAP-expressing reactive astrocytes use MAOB to produce and release excess GABA, causing tonic synaptic inhibition and altered dopamine function [110]. Therefore, the presence of more reactive astrocytes (GFAP) and MAOB in BP and SCHIZ could not only be caused by GABA signaling but contribute to further alterations in regional excitatory/inhibitory balance and dopaminergic dysfunction.

There was decreased expression of the GABA transporter GAT-3 (SLC6A11) in SCHIZ. SLC6A11 is abundantly expressed in astrocytic processes surrounding synapses and neuronal bodies [111, 112]. GAT-3 activity causes extracellular GABA uptake and sodium ion accumulation in astrocytes. Due to sodium/calcium exchange, this leads to increased intracellular astrocytic calcium [111]. Therefore, decreased GAT-3 function in SCHIZ could contribute to excess GABA, but dampen further elevations in calcium due to GABA signaling.

Finally, astrocytic metabolism of GABA is performed by mitochondrial GABA transaminase (ABAT) [113]. ABAT was upregulated in BP and SCHIZ. Increased expression of this degrading enzyme could shorten GABA availability, causing disinhibition. Increased ABAT expression could underlie manic symptoms in BP, as ABAT deficiency was associated with a converse phenotype (inconsolable crying, dullness, lethargy) in case studies [114], and increased ABAT was associated with aggression in rodents [115]. Rare genetic variants in ABAT have been associated with BP [97].

Interestingly, in exploratory analyses, signatures of glial activation (upregulated GFAP) and GABA dysfunction (upregulated SLC6A11 and GABRB1) were strongly associated with substances of abuse, “interactions with the legal system” (GABRB1), and “fatigue” (SLC6A11). This raises the question of whether glial activation and GABA dysfunction in BA10 might be particularly important for the executive dysfunction and cognitive symptoms accompanying BP and SCHIZ. This could complement recent evidence showing elevated GFAP in serum accompanying declines in executive and cognitive function in older individuals [116].

### Limitations and future directions

Differential expression is not always predictive of protein levels, but proteins are challenging to quantify in postmortem tissue due to degradation. Moreover, our qPCR results were derived from gross anatomical samples, and therefore specific cellular relationships cannot be determined. Future endeavors should evaluate mRNA expression in BP and SCHIZ in BA10 using cell-type specific quantitation and high-resolution cortical layer quantitation.

### Conclusion

SCHIZ and BP are associated with notably dysregulated expression of several neurotransmission-related genes in BA10, a cortical area highly specialized in humans. These effects included alterations in gene expression underlying serotonin, dopamine, GABA, and astrocytic networks. We hypothesize that these disruptions underly specific cognitive and behavioral manifestations common to the two disorders.

## Supplementary information

Supplemental Methods and Results

Table S1

Table S2

Table S3

Table S4

Table S5

Table S6

Table S7

Table S8

Table S9

## Data Availability

All analysis code (Rstudio v1.0.153, R v3.4.1) has been released on GitHub (https://github.com/hagenaue/Adriana_FrontalPole, https://github.com/hagenaue/FrontalPole_Microarray).
